# Single-nucleotide resolution of *N*^6^-adenine methylation sites in DNA and RNA by nitrite sequencing†

**DOI:** 10.1039/d0sc03509b

**Published:** 2020-11-05

**Authors:** Yasaman Mahdavi-Amiri, Kimberley Chung Kim Chung, Ryan Hili

**Affiliations:** Department of Chemistry, Centre for Research on Biomolecular Interactions, York University 4700 Keele Street Toronto ON M3J 1P3 Canada rhili@yorku.ca www.yorku.ca/rhili

## Abstract

A single-nucleotide resolution sequencing method of *N*^6^-adenine methylation sites in DNA and RNA is described. Using sodium nitrite under acidic conditions, chemoselective deamination of unmethylated adenines readily occurs, without competing deamination of *N*^6^-adenine sites. The deamination of adenines results in the formation of hypoxanthine bases, which are read by polymerases and reverse transcriptases as guanine; the methylated adenine sites resist deamination and are read as adenine. The approach, when coupled with high-throughput DNA sequencing and mutational analysis, enables the identification of *N*^6^-adenine sites in RNA and DNA within various sequence contexts.

## Abstract 2

Chemoselective deamination of adenine in the presence of *N^6^*-methyladenine using nitrite enables single-nucleotide resolution sequencing of *N*^6^-adenine methylation sites in DNA and RNA.

## Graphical abstract

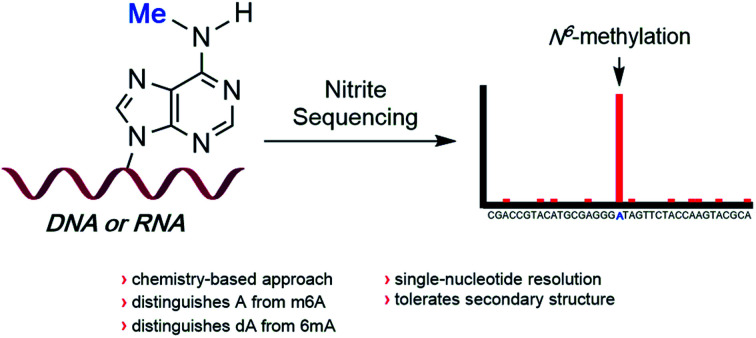

## Introduction

The ability to map methylation sites in the human genome and epitranscriptome has transformed our understanding of how these modifications govern and influence a host of cellular processes and diseases.^1,2^ Amongst the most widely studied methylations is *N*^6^-methyladenine, known as 6mA in DNA and m^6^A in RNA. m^6^A is the most common methylation observed in RNA, where it constitutes 0.1–0.4% of adenosines, and accounts for approximately 50% of total methylations in RNA.^3^ The dynamics of m^6^A incorporation into RNA are regulated by “writers” (*i.e.*, methyltransferases) and “erasers” (*i.e.*, demethyltransferases), and can directly affect processes such as nuclear RNA export, splicing, and RNA stability.^4^ Not surprisingly, the deregulation of these dynamics and resulting aberrant levels of m^6^A has been linked to obesity, immunoregulation, and cancer.^5^ While 6mA has been widely known as a DNA modification in prokaryotes, its presence in eukaryotes has only been recently established, including in humans where it represents ∼0.051% of the genome.^6^ 6mA is thought to play an epigenetic role in embryonic development,^7^ tumorigenesis,^6^ response to stress, neuropsychiatric disorders,^8^ and embryonic stem cell function,^9^ and it can be inherited.^10^

**Fig. 1:**
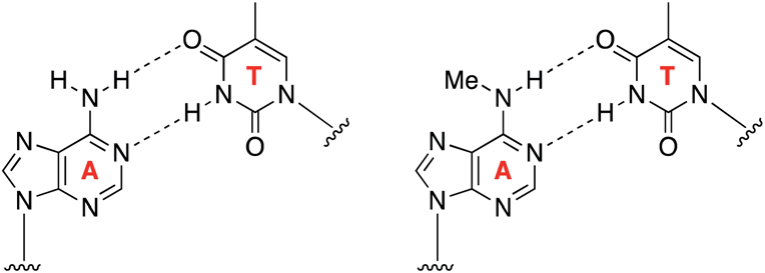
Similar Watson–Crick–Franklin base-pairing observed between adenine and thymine (left) and *N*^6^-methyladenine and thymine (right) limits direct high-throughput sequencing.

Understanding the role of *N*^6^-methyladenine in RNA and DNA requires robust single-nucleotide sequencing methods. Due to the similar Watson–Crick–Franklin hydrogen-bonding nature of adenine and *N*^6^-methyladenine with thymine, direct high-throughput sequencing has been challenging using conventional methods (Fig. 1). This notwithstanding, several existing methods have been developed to probe the m^6^A and 6mA methylomes; however, each of these suffer from limitations. Immunoprecipitation (IP) of short RNA fragments using m^6^A-specific antibodies, MeRIP-seq,^11,12^ followed by sequencing provides low resolution mapping; miCLIP,^13^ which involves the UV-induced cross-linking of the m^6^A antibody to RNA, requires a cytosine residue at the +1-position, rendering a potentially large number of m^6^A sites undetectable; m^6^A-sensitive RNA-endoribonuclease-facilitated sequencing (m^6^A-REF-seq) detects only at the ACA motif, which reduces sequence space; polymerases have also been used to detect m^6^A in RNA by either increased mutation frequency,^14,15^ or decreased rate of incorporation^16^ across from m^6^A; however, these have yet to find wide-scale use, and can give false positives of adenosines that are in close proximity downfield from the m^6^A site.^14^ Similarly, while several 6mA sequencing methods are available, many of them suffer from issues. Traditional IP-based methods, such as 6mA-DIP-seq,^17,18^ suffer from low resolution; IP methods coupled with restriction digest, such as DA-6mA-seq,^19^ improve resolution at the expense of sequence space; PacBio single-molecule real-time (SMRT) sequencing technology,^20^ enhances the resolution down to the single-nucleotide level, but suffers from false positives^21,22^ and struggles with genomes high in 5mC;^21,23^ and 6mA-crosslinking-exonuclease-sequencing (6mACE-seq), enables single-nucleotide resolution, but suffers from an extensive workflow. New single-nucleotide sequencing methods for both m^6^A and 6mA continue to be needed to provide access to probe the complete sequence space of RNA and DNA, enabling in-depth functional studies of these methylomes.

As opposed to enzyme-mediated sequencing methods, chemical reactions are often less sequence dependent, can work on either DNA or RNA, and thus can provide a robust, inexpensive, and universal sequencing approach to probe the 6mA and m^6^A methylomes. To this end, we were inspired by the simplicity of bisulfite sequencing,^24^ which has been extensively used to map the sites of 5-methylcytosine (5mC) residues in DNA and RNA. The method involves the bisulfite-catalysed chemoselective deamination of cytosine resulting in a cytosine to uracil (C → U) transition, while leaving 5mC largely unaffected by the process. Thus, comparative sequencing analysis against a no-reaction control can be used to readily identify the locations of 5mC within a DNA or RNA sequence. We were inspired to use a similar approach to enable the single-nucleotide resolution of m^6^A and 6mA in RNA and DNA, respectively. To achieve this, we required a chemical reaction that (i) was water tolerant; (ii) did not degrade DNA or RNA; (iii) was chemoselective for either *N*^6^-methyladenine or unmethylated adenine; and (iv) resulted in a change in how the nucleobase was read by a polymerase or reverse transcriptase.

**Fig. 2:**
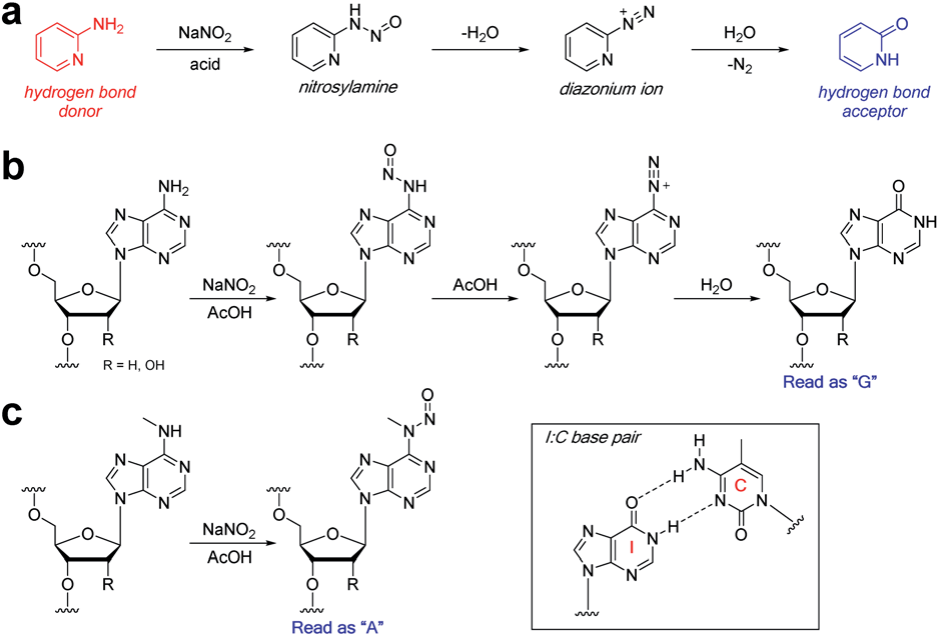
Nitrite reaction with aromatic amines. (a) Reaction of 2-aminopyridine with sodium nitrite under acidic aqueous conditions. Reaction of nitrite with (b) adenine and (c) *N*^6^-methyladenine bases in RNA or DNA. Inset: inosine nucleobases are read as guanine by polymerases.

We were drawn to the nitrite-mediated diazotisation of aromatic amines, first described by Griess,^25^ as a possible reaction that would satisfy our four criteria – in particular the process later described on 2-aminopyridines (Fig. 2a).^26^ In the presence of acid under aqueous conditions, nitrite forms reactive nitrosonium ion, which reacts with aromatic amines to form nitrosamines. Subsequent dehydration to form the diazonium ion can only proceed with primary aromatic amines, as secondary aromatic amines lack the additional N–H required for dehydration. Hydrolysis of the diazonium yields the deaminated product. Accordingly, the process should be chemoselective for the primary exocyclic amine of adenine over the secondary exocyclic amine of *N*^6^-methyladenine seen in m^6^A and 6mA (Fig. 2b and c). Thus, only unmethylated adenine will be hydrolysed under these conditions to form hypoxanthine – an exchange of a hydrogen bond donor for a hydrogen bond acceptor. Polymerases are known to read hypoxanthine as guanine,^27^ resulting in an A → G transition, which can be detected by high-throughput DNA sequencing. Other exocyclic amines in DNA and RNA will also be susceptible to nitrite-mediated deamination, including those on guanine and cytosine, which will result in G → A transitions and C → T/U transitions; however, these can be handled during sequencing data analysis.

## Experimental section

### Chemicals and materials

Unless otherwise noted, water was purified with the Milli-Q Direct Q3. DNA and RNA oligonucleotides were purchased from Integrated DNA Technologies, with HPLC purification.

Nucleoside analysis was performed by reverse-phase high-performance liquid chromatography (HPLC, Agilent 1260 Infinity II) using a C_18_ stationary phase (Phenomenex, Luna® 5 μm C_18_(2) 100 Å, 250 × 4.6 mm) and an acetonitrile/100 mM triethylammonium acetate gradient. Oligonucleotide concentrations were determined by Qubit 4.0 Fluorometer (Thermo Fisher Scientific) using the dsDNA HS Assay Kit (Invitrogen, Q32851). High-throughput DNA sequencing samples were quantified using a Qubit 4 Fluorometer, prepared on an Ion Chef instrument and sequenced on an Ion Torrent GeneStudio S5 Plus using Ion 530 Chips.

### Nitrite-mediated sequencing of DNA

In a PCR tube was added 20 pmol (2 μL, 10 μM) of ssDNA, 12.3 μL Milli-Q water and 0.7 μL acetic acid (Fisher Scientific, A38-212). Then, 15 μL of freshly-prepared 2 M sodium nitrite (Sigma-Aldrich, 237213-5G) was added, mixed thoroughly, and incubated on a thermal cycler (Biorad, T100) at 22 °C for 5 h. The reaction was then purified using E.Z.N.A. Cycle Pure Kit (Omega Bio-tek, D6492). The purified DNA was prepared for sequencing by PCR using IonCode adapters and Q5 High-Fidelity 2× Master Mix (New England Biolabs, M0492) (see ESI† for sequences and PCR protocol).

The amplified DNA was purified using E.Z.N.A. Cycle Pure Kit (Omega Bio-tek, D6492), and then purified using 10% native polyacrylamide gel. After staining the gel for 15 minutes with SYBR safe DNA gel stain (Invitrogen, 33100), the gel was visualised on BluPAD Dual LED Blue/White Light Transilluminator (Bio-helix, BP001CU), and the desired DNA amplicon was excised from the gel. The excised band was crushed into a slurry, 100 μL of 0.3 M NaCl was added to the slurry, and incubated overnight at 37 °C. The DNA was then purified from the slurry using a CENTRI-SEP spin column (Princeton Separation, CS-901) pre-hydrated with Milli-Q water. The concentration of the DNA was measured using a Qubit 4.0 Fluorometer (Thermo Fisher Scientific) using the dsDNA HS Assay Kit (Invitrogen, Q32851) and then diluted to 50 pM. The prepped and pooled DNA libraries were loaded onto an Ion Chef with Ion 530 Chips (Thermo Fisher Scientific, A27764). The prepared chips were then sequenced on an Ion GeneStudio™ S5 Plus DNA sequencing system (Thermo Fisher Scientific).

### Nitrite-mediated sequencing of RNA

In a PCR tube was added 20 pmol (2 μL, 10 μM) of ssRNA, 11.5 μL nuclease free water (Ambion, AM9937) and 1.5 μL acetic acid (Fisher Scientific, A38-212). Then, 15 μL of freshly-prepared 2 M sodium nitrite (Sigma-Aldrich, 237213-5G) was added, mixed thoroughly, and incubated on a thermal cycler (Biorad, T100) at 22 °C for 5 h. The reaction was then purified using Monarch RNA cleanup kit (New England BioLabs, T2030L). The purified RNA was prepared for sequencing by reverse transcription PCR using IonCode adapters and SuperScript III one-step RT-PCR system with Platinum Taq DNA Polymerase (Invitrogen, Thermo Fisher Scientific, 12574-018) (see ESI† for sequences and RT-PCR protocol).

The reverse transcribed DNA was purified using E.Z.N.A. Cycle Pure Kit (Omega Bio-tek, D6492), and then purified using 10% native polyacrylamide gel. After staining the gel for 15 minutes with SYBR safe DNA gel stain (Invitrogen, 33100), the gel was visualised on BluPAD Dual LED Blue/White Light Transilluminator (Bio-helix, BP001CU), and the desired DNA amplicon was excised from the gel. The excised band was crushed into a slurry, 100 μL of 0.3 M NaCl was added to the slurry and incubated overnight at 37 °C. The DNA was then purified from slurry using a CENTRI-SEP spin column (Princeton Separation, CS-901) pre-hydrated with Milli-Q water. The concentration of the DNA was then measured using a Qubit 4.0 Fluorometer (Thermo Fisher Scientific) using the dsDNA HS Assay Kit (Invitrogen, Q32851) and then diluted to 50 pM. The prepped and pooled DNA libraries were loaded onto an Ion Chef with Ion 530 Chips (Thermo Fisher Scientific, A27764). The prepared chips were then sequenced on an Ion GeneStudio™ S5 Plus DNA sequencing system (Thermo Fisher Scientific).

### Sequencing analysis

FastQ files generated from the Ion Torrent system were trimmed and processed for quality using the single-end read function in Trimmomatic 0.36.^28^ Bowtie 1 (ref. 29) was used to build the template index and generate the map file for each experiment. Map files were analysed for transitions and transversion at each nucleobase. Graphs were plotted from each adenosine as the ratio of the frequency of (d)A → (d)G transitions for the demethylated experiment over the frequency of (d)A → (d)G transitions for the methylated experiment.

## Results and discussion

### Nitrite-mediated deamination on single nucleosides

**Fig. 3:**
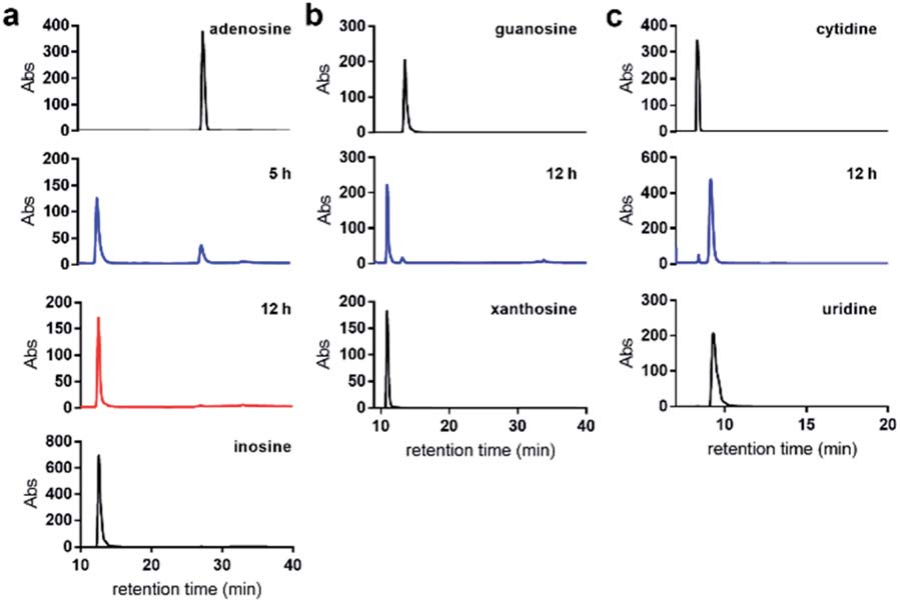
Nitrite-mediated deamination of free nucleosides. HPLC analysis of the conversion of (a) adenosine into inosine; (b) guanosine into xanthosine; and (c) cytidine into uridine using NaNO_2_ and 1.7% aqueous AcOH, at 22 °C over 12 h.

We first examined the nitrite-mediated deamination process on free adenosine. Using a 1 M aqueous NaNO_2_ in the presence of 1.7% AcOH at 22 °C, complete consumption of adenosine into inosine was observed by HPLC analysis over a 12 h period (Fig. 3a). Deamination of guanosine into xanthosine (Fig. 3b) and cytidine into uridine (Fig. 3c) was largely completed over a 12 h period under similar conditions. This suggests that nitrosylation and subsequent diazotisation of adenosine could be achieved using conditions that are compatible with nucleic acids. We observed that deamination of adenosine into inosine was over 50% completed within 5 h. In order not to scramble the alignment of DNA and RNA sequences against a genome, we decided that 5 h would be sufficient for detecting difference in deamination at methylated sites.

**Fig. 4:**
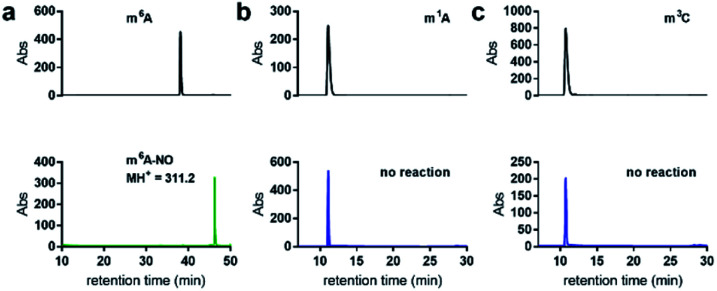
Nitrite-mediated deamination of methylated nucleosides. (a) HPLC analysis of the conversion of m^6^A into nitrosylated m^6^A (m^6^A-NO) using NaNO_2_ and 1.7% aqueous AcOH, at 22 °C over 3.5 h. m^6^A-NO was confirmed by ESI-MS. Note that inosine was not detected by HPLC analysis of nitrosylation of m^6^A. No reaction was observed over a 12 h period for (b) m^1^A or (c) m^3^C.

When subjecting *N*^6^-methyladenosine to the same conditions, full conversion into *N*^6^-nitroso-m^6^A was observed within 3.5 h, with no trace amounts of inosine formed over a 12 h period (Fig. 4a). The lack of conversion of m^6^A-NO into inosine highlights the resistance to hydrolysis under the tested experimental conditions. Interestingly, m^6^A becomes nitrosylated significantly faster than adenosine owing to its increased nucleophilicity at the *N*^6^ position. Other examined methylated nucleosides, including m^1^A (Fig. 4b) and m^3^C (Fig. 4c) were unreactive under the tested conditions. This is due to the decrease in electron density of these positively charged nucleobases.^30^

### Optimisation of nitrite-mediated deamination on DNA and RNA

**Fig. 5:**
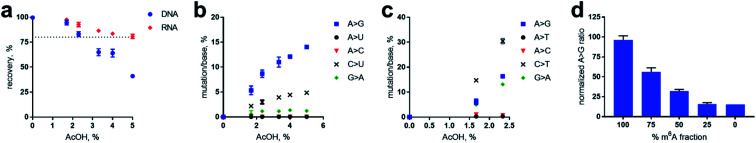
Optimisation of nitrite-mediated deamination on RNA and DNA. “>” denotes corresponding transition or transversion. (a) Recovery of DNA and RNA with respect to acid concentration during the nitrite reaction. Error based on assessment in duplicates. Dotted line represents 80% threshold of recovery. (b) High-throughput sequencing of RNA after nitrite reaction at varying acid concentrations. Mutations are represented in legend, and correspond to the specific type of mutation per expected nucleobase. (c) High-throughput sequencing of DNA after nitrite reaction at varying acid concentrations. Note that high-throughput DNA analysis above 2.3% AcOH was not processed due to undesirably low isolation (per Fig. 4a). (d) Quantification of methylation fraction of an adenosine site within an RNA sequence. See ESI† for sequences.

Prior to evaluating the performance of the nitrite-mediated deamination process on sequencing, we determined the stability of RNA and DNA in the reaction conditions while optimising variables. We found that acid had the most profound effect on the stability of RNA and DNA during the process. Using a ssDNA and ssRNA as models (see ESI† for sequence information), we monitored the degradation of the sequences with increasing acid concentration using 1 M NaNO_2_ for 5 h at 22 °C (Fig. 5a). We observed that DNA was far more sensitive than RNA under the acidic conditions used. We attributed the degradation due to acid-catalysed depurination and backbone cleavage, albeit cationic intermediates during the diazotisation process could also play a role. RNA, with its electronegative 2′-OH group is less susceptible to this depurination/cleavage process.^31,32^ To facilitate isolation and the study of low amounts of DNA and RNA, we decided to place an 80% recovery threshold on the process, which limited acid concentration for RNA to 5% and DNA to 2.3%.

We next sought to study and optimise the A → G transition reaction on a model 60 nt RNA sequence containing one instance of m^6^A. We subjected the sequence to 1 M NaNO_2_ for 5 h at 22 °C with acetic acid concentrations ranging from 0 to 5%. As anticipated, we observed that increasing the percentage of AcOH increased the A → G transitions from background error rates of less than 0.1% transitions per adenosine to 14% when using 5% AcOH (Fig. 5b), which is attributed to acid-promoted increase in nitrosonium ion concentration. Importantly, these data demonstrate no change in the frequency of A → C and A → U transversions caused by the reaction. As expected, deamination at cytosine and guanosine was observed, resulting in C → U and G → A mutations (Fig. 5b). Fortuitously, nitrosylated m^6^A was read as adenosine by reverse transcriptase, and had a similar frequency of A → G transitions from adenosines in the no-reaction control. This result was unexpected due to the loss of canonical hydrogen-bonding to thymine during reverse transcription; however, alternative non-canonical interaction with thymine might be at play that give preference to thymine incorporation.

Due to the lower stability of DNA under the AcOH-promoted nitrite reaction, we examined only those acid concentrations yielding >80% recovery. Similar to the RNA experiments, increasing mutation frequencies of dA → dG, dC → dT, and dG → dA were observed with increasing AcOH concentrations (Fig. 5c). Curiously, dC → dT mutations were greater than those of dA → dG – the opposite of which was observed in RNA (Fig. 5b). The higher propensity for deamination of cytosine in DNA over that of RNA has been previously observed in activation-induced deaminase processing of nucleic acids.^33^ The increase in dG → dA mutation in DNA over RNA is unclear, and compounded by the fact that deamination of the adenine base results in xanthine, which may be read with different error frequencies and propensities by DNA polymerases and reverse transcriptases. After concluding the optimisation studies, we found that the recovery boundary concentrations of 5% AcOH for RNA and 2.3% AcOH for DNA represented the best conditions for deamination activity. While, in principle, these mutations could be increased by further optimisation, we chose not to push the process too far so as to avoid issues in sequence alignment during high-throughput sequencing analysis.

### Evaluation of nitrite-mediated sequencing of *N*^6^-methyladenine sites in DNA and RNA

**Fig. 6:**
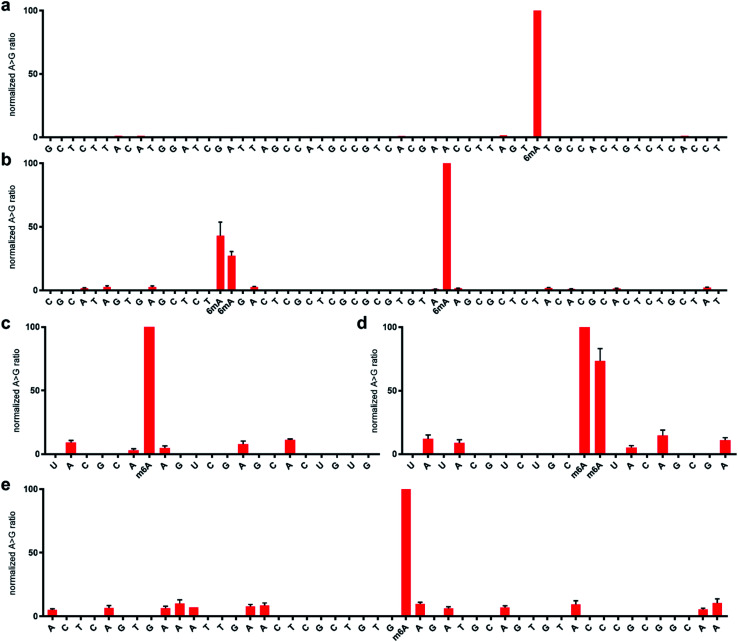
Normalised sequencing representation of the ratio of (d)A → (d)G mutation at each nucleobase following treatment with 1 M sodium nitrite in the presence of acetic acid for 5 h at 22 °C. The DNA sequences contain a single 6mA site at position 63 (a) and three 6mA sites at positions 35, 36, and 55 (b). The RNA sequences contain a single m^6^A site at position 26 (c) and two m^6^A sites at positions 31 and 32 (d). The 23S rRNA from *E. coli* contains a single m^6^A site at position 2030 (e). Primer sequence regions are not shown for clarity. See ESI† for complete sequences and predicted folded structures determined with 1 M Na^+^ at 22 °C using MFold.

With the optimised system in hand, we examined the sequencing method for its ability to detect *N*^6^-methyladenine within DNA and RNA. A 99 nt DNA sequence containing a single 6mA site was incubated with 1 M NaNO_2_ and 2.3% aqueous acetic acid, and subsequently analysed by high-throughput DNA sequencing, trimmed for length and quality, and aligned to the reference sequence using bowtie 1 to enable induced SNP calling.^29^ The demethylated sequence was also subjected to the same process for comparative analysis. As expected, extensive deamination was observed, with dA → dG transitions increasing >50-fold against the no-reaction control. We plotted the normalised ratio (*R*) of the dA → dG transitions at each nucleotide position compared to that of the demethylated sequence:
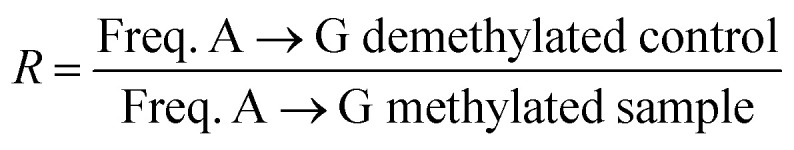


This afforded a convenient way to visualise the nitrite sequencing data (Fig. 6a). High A → G transition ratios are observed only at the 6mA sites, which is consistent with the nucleoside reaction data. Encouraged by these findings, we attempted 6mA sequencing on a more challenging template – one comprising two dAs flanking a 6mA site, and also a double 6mA site, which would be overlooked by most existing sequencing methods should such motifs occur in nature. The method readily detected the flanked 6mA site, highlighting the single-nucleotide resolution (Fig. 6b). The contiguous 6mA sites were more challenging, yet still distinguished from unmethylated adenine sites. This slightly lower response may be due to neighbouring group effects during diazotisations of adjacent nitrosylated adenines. The method was also compatible with duplex DNA and readily detected 6mA sites (Fig. S1†), albeit with an expected decrease in response likely resulting from amplification of the non-target strand.

We next explored the nitrite sequencing method to detect m^6^A in RNA using similar conditions as those used for DNA. One 60 nt sequence comprised a single m^6^A flanked by two adenosines, which yielded good differentiation amongst other adenosines in the sequence (Fig. 6c), again highlighting the single-nucleotide discrimination of the nitrite sequencing method. We also attempted the sequencing method on a contiguous instance of m^6^A within a 60 nt RNA. Good detection above background was observed (Fig. 6d); however, issues with potential neighbouring group interference of nitrosylation were similarly noted. Due to the importance of quantifying the methylation fraction at potential m^6^A sites, we performed a spike-in experiment that assessed the response for varying fractions of m^6^A at a specific adenosine site in RNA. We found that the nitrite sequencing method was able to quantify m^6^A fractions down to 50%, below which the response was not significant above background levels (Fig. 5d). We further sought to apply the sequencing method to detect naturally occurring m^6^A in isolated RNA. To this end, *E. coli* rRNA, which is known to have an m^6^A site at position 2030 of the 23S subunit,^34^ was purified and subjected to nitrite sequencing (Fig. 6e). The m^6^A site at position 2030 was readily detected, with approximately 10-fold increase in signal over neighbouring unmodified adenosines. We observed that peptides interfered with the desired nitrite chemistry on RNA, and thus should be thoroughly removed from samples.

In all sequencing experiments, we observed slightly higher background noise with RNA nitrite sequencing compared with that of DNA. This could potentially be related to greater folding of single-stranded RNA *versus* DNA. Potential avenues around this would be the addition of mild denaturants and solvents. Such optimizations may also boost the quantification range for the level of methylation at putative m^6^A sites and enable detection of low abundance m^6^A sites in biological samples. These approaches are currently being investigated.

### Potential applications and limitations of nitrite sequencing toward other modifications

The nitrite-mediated deamination process on DNA and RNA is anticipated to have further applications but also limitations in resolving other related methylation and alkylation sites. For instance, *N*^6^,2′-*O*-dimethyladenosine (m^6^Am), which is located in certain RNA transcripts at the first position following the 7-methylguanosine cap, would also not be able to undergo deamination in the presence of nitrite, resulting in a high *R* value similar to m^6^A. While this could potentially yield false positives for m^6^A sequencing, m^6^Am is primarily located at the adenosine of the first encoded nucleotide in mRNA and could be handled through post-sequencing analysis. Furthermore, in principle, the nitrite sequencing method could be used to identify such m^6^Am sites in transcripts. We have also identified other common modified nucleosides that would give high *R* values during sequencing analysis. m^1^A and m^3^C, both of which are too electron poor to react with nitrosonium ion under the examined conditions (Fig. 4b and c), do not deaminate, thus this method could potentially be used for m^1^A and m^3^C sequencing to complement other burgeoning methods.^35^

## Conclusions

In conclusion, we have demonstrated the first chemistry-based method to facilitate the sequencing of both m^6^A in RNA and 6mA in DNA. The chemistry takes advantage of the acid-mediated nitrite reaction that chemoselectively deaminates adenine in the presence of *N*^6^-methyladenine. This results in a large increase in (d)A → (d)G transitions only at unmethylated sites. When coupled to high-throughput DNA sequencing, nitrite sequencing enables the identification of m^6^A and 6mA sites at single-nucleotide resolution. We anticipate that this sequencing method will find broad use as a straightforward and affordable approach to detect *N*^6^-adenine methylation sites in RNA and DNA.

## Conflicts of interest

There are no conflicts to declare.

## Supplementary Material

SC-012-D0SC03509B-s001
