# Supplementary material for: Single-nucleotide resolution of N6-adenine methylation sites in DNA and RNA by nitrite sequencing
Source: Chem Sci. 2020 Nov 5;12(2):606–12. doi: 10.1039/d0sc03509b (PMC8179008; doi:10.1039/d0sc03509b)
Supplement: SC-012-D0SC03509B-s001 [file SC-012-D0SC03509B-s001.pdf]

Electronic Supplementary Material (ESI) for Chemical Science.
This journal is © The Royal Society of Chemistry 2020

Supporting Information

Single-Nucleotide Resolution of N^6-Adenine Methylation Sites in DNA and RNA by Nitrite Sequencing

Yasaman Mahdavi-Amiri, Kimberley Chung Kim Chung, and Ryan Hili*

[†]Department of Chemistry, Centre for Research on Biomolecular Interactions, York University, Toronto, Ontario M3J 1P3, Canada.

Table of Contents

Complete References 2
Supporting Methods 2
- General Information 2
- DNA Sequences 2
- RNA Sequences 3
- IonCode adapters 3
- Nitrite reaction on nucleosides 4
- HPLC analysis 4
- Nitrite sequencing for DNA 5
- Nitrite sequencing for RNA 6
- Nitrite sequencing of E. coli 23S rRNA 6
- Sequencing analysis 7

Supporting Data 8
- Example of sequencing data and analysis 8
- Predicted secondary structures 9

Complete References

C. L. Xiao, S. Zhu, M. He, D. Chen, Q. Zhang, Y. Chen, G. Yu, J. Liu, S. Q. Xie, F. Luo, Z. Liang, D. P. Wang, X. C. Bo, X. F. Gu, K. Wang, G. -R. Yan *Mol. Cell.* **2018**, *71*, 306–318.

J. Liu, Y. Zhu, G. -Z. Luo, X. Wang, Y. Yue, X. Wang, X. Zong, K. Chen, H. Yin, Y. Fu, D. Han, Y. Wang, D. Chen, C. He *Nat. Commun.* **2016**, *7*, 13052–13058.

C. Ma, R. Niu, T. Huang, L. W. Shao, Y. Peng, W. Ding, Y. Wang, G. Jia, C. He, C. Y. Li, A. He, Y. Liu *Nat. Chem. Biol.* **2019**, *21*, 319–327.

S. Wang, J. Wang, X. Zhang, B. Fu, Y. Song, P. Ma, K. Gu, X. Zhou, X. Zhang, T. Tian, X. Zhou *Chem. Sci.* **2016**, *7*, 1440–1446.

G. Zhang, H. Huang, D. Liu, Y. Cheng, X. Liu, W. Zhang, R. Yin, D. Zhang, P. Zhang, J. Liu, C. Li, B. Liu, Y. Luo, Y. Zhu, N. Zhang, S. He, C. He, H. Wang, D. Chen *Cell*, **2015**, *161*, 893–906.

E. E. Schadt, O. Banerjee, G. Fang, Z. Feng, W. H. Wong, X. Zhang, A. Kislyuk, T. A. Clark, K. Luong, A. Keren-Paz, A. Chess, V. Kumar, A. Chen-Plotkin, N. Sondheimer, J. Korlach, A. Kasarskis *Genome Res.* **2013**, *23*, 129–141.

Supporting Methods

General Information

Unless otherwise noted, water was purified with the MilliQ Direct Q3. DNA and RNA oligonucleotides were purchased from Integrated DNA Technologies, with HPLC purification. Nucleoside analysis was performed by reverse-phase high-performance liquid chromatography (HPLC, Agilent 1260 Infinity II) using a C18 stationary phase (Phenomenex, Luna® 5 µm C18(2) 100 Å, 250 x 4.6 mm) and an acetonitrile/100 mM triethylammonium acetate gradient. Oligonucleotide concentrations were determined using a Qubit 4.0 Fluorometer. High-throughput DNA sequencing samples were quantified using a Qubit 4 Fluorometer, prepared on an IonChef instrument and sequenced on an Ion Torrent GeneStudio S5 Plus using Ion 530 Chips.

DNA Sequences

Note: /iN6Me-dA/ = N6-methyldeoxyadenosine

temp1_unmeth GCT AGT TAT TGC TCA GCG GGC TCT TAC ATG GAT CGA TTA GCC ATG CCG TCA CGA ACC TTA GTA TGC CAC TGT CTC ACC TCC CTA TAG TGA GTC GTA TTA

temp2_unmeth GCT AGT TAT TGC TCA GCG GCG CAT AGT GAG CTC TAA GAC TCG CTC GCG CGT GTA AAG CGC TCT ACA CGC ACT CTG CTA TCC CTA TAG TGA GTC GTA TTA

temp1_6mA_pos63 GCT AGT TAT TGC TCA GCG GGC TCT TAC ATG GAT CGA TTA GCC ATG CCG TCA CGA ACC TTA GT/iN6MedA/ TGC CAC TGT CTC ACC TCC CTA TAG TGA GTC GTA TTA

temp2_6mA_pos35-36-55 GCT AGT TAT TGC TCA GCG GCG CAT AGT GAG CTC T/iN6Me-dA//iN6Me-dA/ GAC TCG CTC GCG CGT GTA /iN6Me-dA/AG CGC TCT ACA CGC ACT CTG CTA TCC CTA TAG TGA GTC GTA TTA

RNA Sequences

Note: /iN6Me-rA/=N6-methyladenosine

temp3_unmeth rGrCrU rArGrU rUrArU rUrGrC rUrCrA rGrCrG rGrUrA rUrArC rGrUrC rUrGrC rArArU rArCrA rGrCrG rArCrC rCrUrA rUrArG rUrGrA rGrUrC rGrUrA rUrUrA

temp4_unmeth rGrCrU rArGrU rUrArU rUrGrC rUrCrA rGrCrG rGrUrA rCrGrC rArArA rGrUrC rGrArG rCrArC rUrGrU rGrCrC rCrUrA rUrArG rUrGrA rGrUrC rGrUrA rUrUrA

temp3_m6A_pos31-32 rGrCrU rArGrU rUrArU rUrGrC rUrCrA rGrCrG rGrUrA rUrArC rGrUrC rUrGrC /iN6Me-rA//iN6Me-rA/rU rArCrA rGrCrG rArCrC rCrUrA rUrArG rUrGrA rGrUrC rGrUrA rUrUrA

temp4_m6A_pos26 rGrCrU rArGrU rUrArU rUrGrC rUrCrA rGrCrG rGrUrA rCrGrC rA/iN6Me-rA/rA rGrUrC rGrArG rCrArC rUrGrU rGrCrC rCrUrA rUrArG rUrGrA rGrUrC rGrUrA rUrUrA

IonCode adapters

T7_P1 CCA CTA CGC CTC CGC TTT CCT CTC TAT GGG CAG TCG GTG ATT AAT ACG ACT CAC TAT AGG G

T7_term_IC_0101 CCA TCT CAT CCC TGC GTG TCT CCG ACT CAG CTAAGGTAAC GGTGAT GCT AGT TAT TGC TCA GCG G

T7_term_IC_0102 CCA TCT CAT CCC TGC GTG TCT CCG ACT CAG TAAGGAGAAC GGTGAT GCT AGT TAT TGC TCA GCG G

T7_term_IC_0103 CCA TCT CAT CCC TGC GTG TCT CCG ACT CAG AAGAGGATTC GGTGAT GCT AGT TAT TGC TCA GCG G

T7_term_IC_0104 CCA TCT CAT CCC TGC GTG TCT CCG ACT CAG TACCAAGATC GGTGAT GCT AGT TAT TGC TCA GCG G

T7_term_IC_0105 CCA TCT CAT CCC TGC GTG TCT CCG ACT CAG CAGAAGGAAC GGTGAT GCT AGT TAT TGC TCA GCG G

23S_+2004_IC_0106 CCA TCT CAT CCC TGC GTG TCT CCG ACT CAG CTGCAAGTTC GGTGAT AGG CTG TCT CCA CCC GAG

23S_+2004_IC_0108 CCA TCT CAT CCC TGC GTG TCT CCG ACT CAG TTCCGATAAC GGTGAT AGG CTG TCT CCA CCC GAG

23S_rRNA_-2053_P1 CCA CTA CGC CTC CGC TTT CCT CTC TAT GGG CAG TCG GTG ATG TTC ACG GGG TCT TTC CGT C

Nitrite reaction on nucleosides

Reaction on adenosine:
In a PCR tube was added 6 µL of 1 mM Adenosine (Sigma-Aldrich, A9251-1G), 37.5 µL milli-Q water and 1.5 µL acetic acid (Fisher Scientific, A38-212). Then, 45 µL of freshly prepared 2 M sodium nitrite (Sigma-Aldrich, 237213-5G) was added, mixed thoroughly, and incubated on a thermal cycler (Biorad, T100) at 22 °C. After 12 h incubation, 10 µL of 2M TEAA was added to the reaction mixture and the resulting 100 µL solution was injected to HPLC.

Reaction on N^6-methyladenosine:
In a PCR tube was added 6 µL of 1 mM N^6-methyladenosine (Carbosynth, NM32281), 37.5 µL milli-Q water and 1.5 µL acetic acid (Fisher Scientific, A38-212). Then, 45 µL of freshly prepared 2 M sodium nitrite (Sigma-Aldrich, 237213-5G) was added, mixed thoroughly, and incubated on a thermal cycler (Biorad, T100) at 22 °C. After 5 h incubation, 10 µL of 2M TEAA was added to the reaction mixture and the resulting 100 µL solution was injected to HPLC.

HPLC analysis

High performance liquid chromatography (HPLC) analyses were conducted on a 1260 Infinity II LC System from Agilent.

HPLC method:

flow rate: 0.5 mL/min
Detection wavelength: 260 nm

mobile phase A: 3% acetonitrile in 0.1 M triethylammonium acetate (92:5:3 deionized water: 2 M TEAA:acetonitrile)

mobile phase B: 90% acetonitrile (9:1 acetonitrile:water)

Elapsed time (min)	%B
0	0
15	0
35	10
65	100
70	100
72	0
85	0

Column: Phenomenex, Luna® 5 µm C18(2) 100 Å, 250 x 4.6 mm

Nitrite sequencing for DNA

In a PCR tube was added 20 pmol (2 µL, 10 µM) of ssDNA, 12.3 µL milli-Q water and 0.7 µL acetic acid (Fisher Scientific, A38-212). Then, 15 µL of freshly-prepared 2 M sodium nitrite (Sigma-Aldrich, 237213-5G) was added, mixed thoroughly, and incubated on a thermal cycler (Biorad, T100) at 22 °C for 5 h. The reaction was then purified using E.Z.N.A. Cycle Pure Kit (Omega Bio-tek, D6492). The purified DNA was prepared for sequencing by PCR using IonCode adapters and Q5 High-Fidelity 2× Master Mix (New England Biolabs, M0492) using the following PCR cycles:

1. 95 °C, 30 s
2. 95 °C, 30 s
3. 50 °C, 30 s
4. 68 °C, 60 s
5. GOTO step 2 (variable)
6. 68 °C, 5 min
7. 4 °C, ∞

The amplified DNA was purified using E.Z.N.A. Cycle Pure Kit (Omega Bio-tek, D6492), and then purified using 10% native polyacrylamide gel. After staining the gel for 15 minutes with SYBR safe DNA gel stain (Invitrogen, 33100), the gel was visualized on BluPAD Dual LED Blue/White Light Transilluminator (bio-helix, BP001CU), and the desired DNA amplicon was excised from the gel. The excised band was crushed into a slurry, 100 µL of 0.3 M NaCl was added to the slurry and incubated overnight at 37 °C. The DNA was then purified from slurry using a CENTRI-SEP spin column (Princeton Separation, CS-901) pre-hydrated with milli-Q water. The concentration of the DNA was then measured using a Qubit 4.0 Fluorometer (Thermo Fisher Scientific) using the dsDNA HS Assay Kit (Invitrogen, Q32851) and then diluted to 50 pM. The prepped and pooled DNA libraries were loaded onto an Ion Chef with Ion 530 Chips (Thermo Fisher Scientific, A27764). The prepared chips were then sequenced on an Ion GeneStudio™ S5 Plus DNA sequencing system (Thermo Fisher Scientific).

Nitrite sequencing for RNA

In a PCR tube was added 20 pmol (2 µL, 10 µM) of ssRNA, 11.5 µL nuclease free water (Ambion, AM9937) and 1.5 µL acetic acid (Fisher Scientific, A38-212). Then, 15 µL of freshly-prepared 2 M sodium nitrite (Sigma-Aldrich, 237213-5G) was added, mixed thoroughly, and incubated on a thermal cycler (Biorad, T100) at 22 °C for 5 h. The reaction was then purified using Monarch RNA cleanup kit (NEW ENGLAND BioLabs, T2030L). The purified RNA was prepared for sequencing by reverse transcription PCR using IonCode adapters and SuperScript III One-Step RT-PCR System with Platinum *Taq* DNA Polymerase (Invitrogen, Thermo Fisher Scientific, 12574-018) using the following PCR cycles:

1. 55 ^{o}C, 30 min
2. 94 ^{o}C, 2 min
3. 94 ^{o}C, 15 s
4. 50 ^{o}C, 30 s
5. 68 ^{o}C, 60 s
6. GOTO step 3 (9X)
7. 68 ^{o}C, 5 min
8. 4 ^{o}C, ∞

The reverse transcribed DNA was purified using E.Z.N.A. Cycle Pure Kit (Omega Bio-tek, D6492), and then purified using 10% native polyacrylamide gel. After staining the gel for 15 minutes with SYBR safe DNA gel stain (Invitrogen, 33100), the gel was visualized on BluPAD Dual LED Blue/White Light Transilluminator (bio-helix, BP001CU), and the desired DNA amplicon was excised from the gel. The excised band was crushed into a slurry, 100 µL of 0.3 M NaCl was added to the slurry and incubated overnight at 37 °C. The DNA was then purified from slurry using a CENTRI-SEP spin column (Princeton Separation, CS-901) pre-hydrated with milli-Q water. The concentration of the DNA was then measured using a Qubit 4.0 Fluorometer (Thermo Fisher Scientific) using the dsDNA HS Assay Kit (Invitrogen, Q32851) and then diluted to 50 pM. The prepped and pooled DNA libraries were loaded onto an Ion Chef with Ion 530 Chips (Thermo Fisher Scientific, A27764). The prepared chips were then sequenced on an Ion GeneStudio™ S5 Plus DNA sequencing system (Thermo Fisher Scientific).

Nitrite sequencing of E. coli 23S rRNA

rRNA was isolated from *E. coli* ribosome (NEW ENGLAND BioLabs, P0763S) using Monarch RNA cleanup kit (NEW ENGLAND BioLabs, T2030L) to remove peptide fragments. In a PCR tube was added 20 pmol (2 µL, 10 µM) of rRNA and 11.5 µL nuclease free water (Ambion, AM9937). The sample was denatured at 70 ^{o}C for 5 minutes and snap-cooled on ice. Then 1.5 µL acetic acid (Fisher Scientific, A38-212) and 15 µL of freshly-prepared 2 M sodium nitrite (Sigma-Aldrich, 237213-5G) were added, mixed thoroughly, and incubated on a thermal cycler (Biorad, T100) at 22 ^{o}C for 5 h. The reaction was then purified using Monarch RNA cleanup kit (NEW ENGLAND BioLabs, T2030L). The purified RNA was prepared for sequencing by reverse transcription PCR using IonCode adapters and SuperScript III One-Step RT-PCR System with Platinum Taq DNA Polymerase (Invitrogen, Thermo Fisher Scientific, 12574-018) using the following PCR cycles:

1. 55 ^{o}C, 30 min
2. 94 ^{o}C, 2 min
3. 94 ^{o}C, 15 s
4. 50 ^{o}C, 30 s
5. 68 ^{o}C, 60 s
6. GOTO step 3 (12X)
7. 68 ^{o}C, 5 min
8. 4 ^{o}C, ∞

The reverse transcribed DNA was purified using E.Z.N.A. Cycle Pure Kit (Omega Bio-tek, D6492), and then purified using 10% native polyacrylamide gel. After staining the gel for 15 minutes with SYBR safe DNA gel stain (Invitrogen, 33100), the gel was visualized on BluPAD Dual LED Blue/White Light Transilluminator (bio-helix, BP001CU), and the desired DNA amplicon was excised from the gel. The excised band was crushed into a slurry, 100 µL of 0.3 M NaCl was added to the slurry and incubated overnight at 37 °C. The DNA was then purified from slurry using a CENTRI-SEP spin column (Princeton Separation, CS-901) pre-hydrated with milli-Q water.

Demethylation of rRNA

In a PCR tube was added 3 µL of 13.3 µM Ribosome (40 pmol), 11.64 µL nuclease free water and incubated at 70 ^{o}C for 5 minutes, then snap-cooled on ice. 20 µL of *2X FTO buffer and 5.36 µL of 22.4 µM FTO (CAYMAN CHEMICAL, 26340) was added and the mixture was incubated at 37 ^{o}C for 4 hours. The reaction was then purified using Monarch RNA cleanup kit (NEW ENGLAND BioLabs, T2030L). The purified RNA was then used for nitrite sequencing.

*2X FTO Buffer: 100 mM HEPES (Fisher Scientific, BP310) [pH 7.5], 600 µM α-Ketoglutaric acid (Sigma-Aldrich, 75890) , 600 µM ammonium iron (II) sulfate hexahydrate (Sigma-Aldrich, 09719), and 4 mM L-Ascorbic acid (SIGMA Life Science, 95209)

Sequencing analysis

FastQ files generated from the IonTorrent system were trimmed to the expected length using the single-end read function Trimmomatic 0.36.[1] Bowtie 1[2] was used to build the template index and generate the map file for each experiment. Map files were analyzed for transitions and transversion at each nucleobase. Graphs were plotted from each adenosine as the ratio of the frequency of A→G transitions for the demethylated experiment over the frequency of A→G transitions for the methylated experiment.

Supporting Data

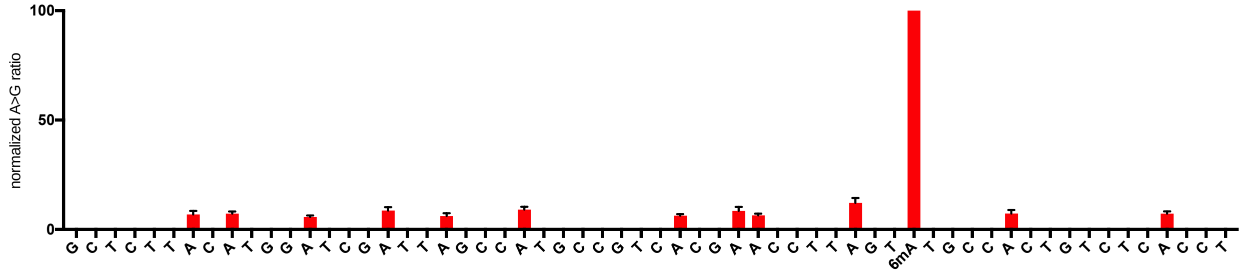

Figure S1. Nitrite sequencing of dsDNA containing a single 6mA site. Duplex DNA was generated from temp1_6mA_pos63 using Klenow extension.

Example of sequencing data and analysis

Sequence: GCT AGT TAT TGC TCA GCG GGC TCT TAC ATG GAT CGA TTA GCC ATG CCG TCA CGA ACC TTA GT/iN6MedA/ TGC CAC TGT CTC ACC TCC CTA TAG TGA GTC GTA TTA

Processed using mapping by trimmomatic 0.36 and Bowtie 1 as described in sequencing analysis section.[2]

Position (A)	A>G (methylated)	freq A>G (methylated)	A>G (demethylated)	freq A>G (demethylated)	ratio
26	577	0.008133634	181	0.002110221	**0.259444**
28	791	0.011150268	251	0.002926329	**0.262445**
32	1009	0.014223287	251	0.002926329	**0.205742**
36	2496	0.035184663	537	0.006260711	**0.177939**
39	241	0.003397237	47	0.000547958	**0.161295**
43	789	0.011122075	190	0.002215149	**0.199167**
51	772	0.010882436	207	0.002413347	**0.221765**
54	388	0.005469411	83	0.00096767	**0.176924**
55	1969	0.02775585	400	0.004663472	**0.168018**
60	426	0.006005075	198	0.002308419	**0.384411**
63	13	0.000183253	332	0.003870682	**21.12201**
68	1319	0.018593177	294	0.003427652	**0.18435**
76	449	0.006329292	143	0.001667191	**0.263409**

Predicted secondary structures

Secondary structures of full DNA and RNA sequences were determined by MFold.[3]
Constraints:
Na^+ = 1 M (to simulated 1 M $NaNO_2$)
T = 22 °C

Temp1

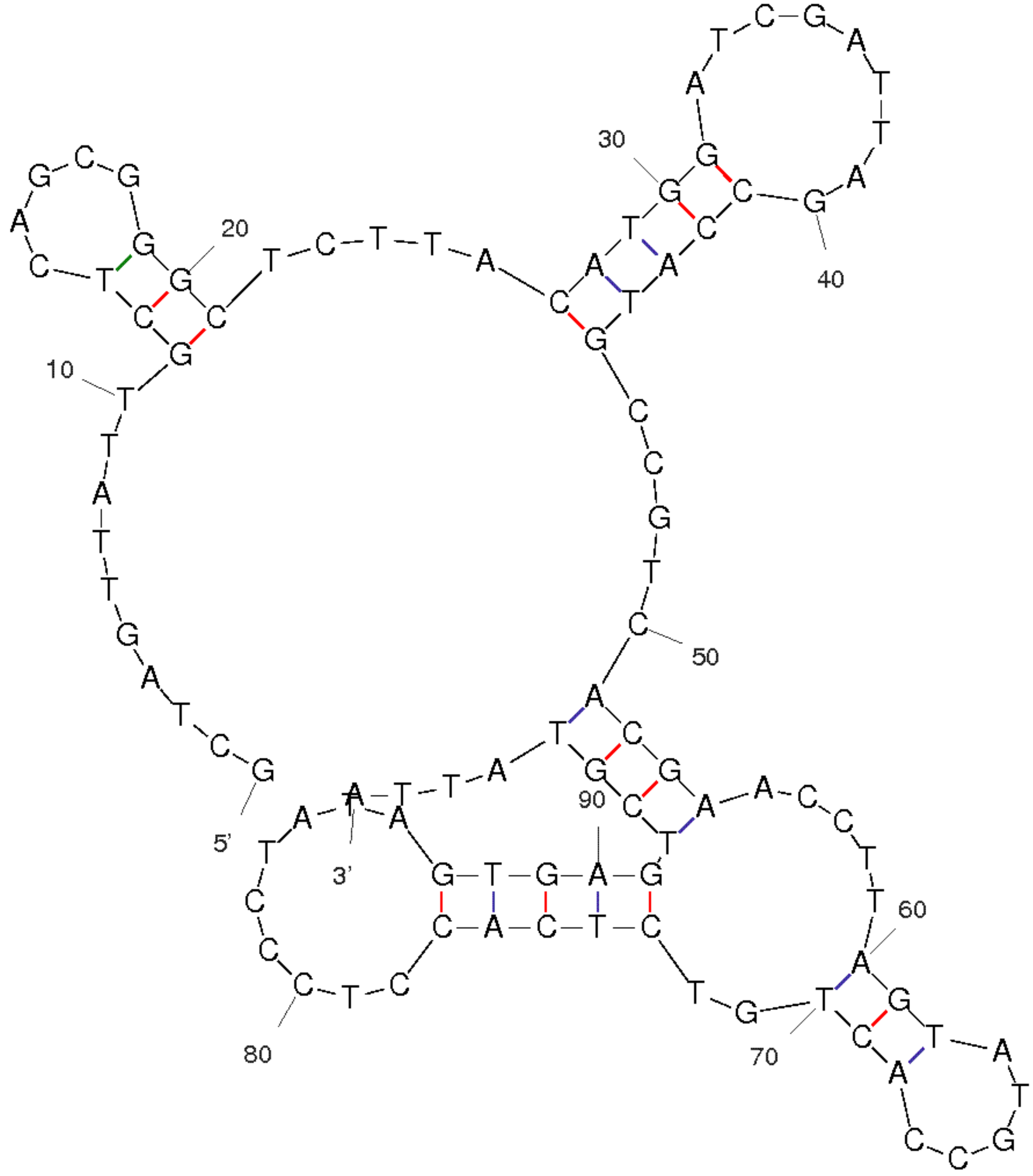

Temp2

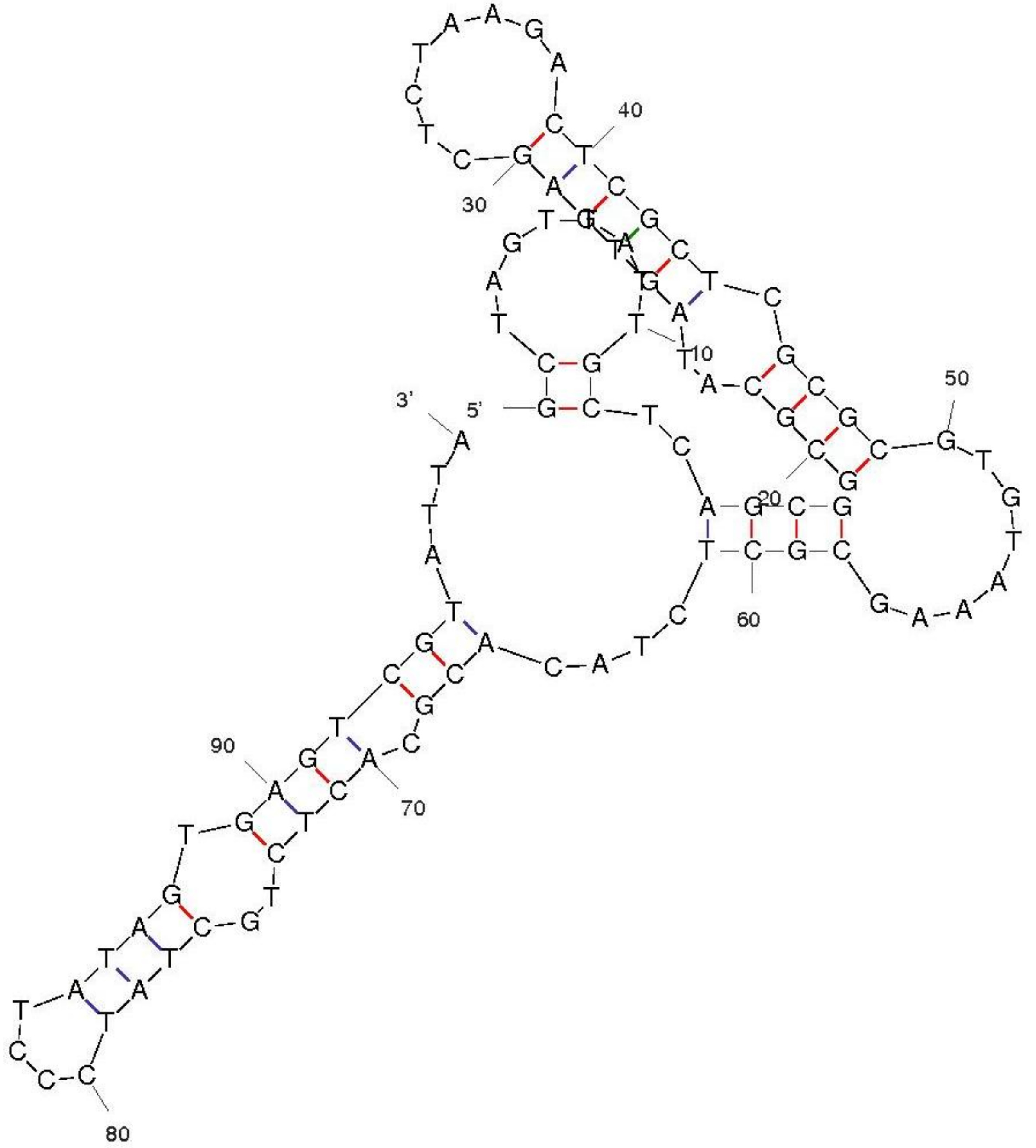

Temp3

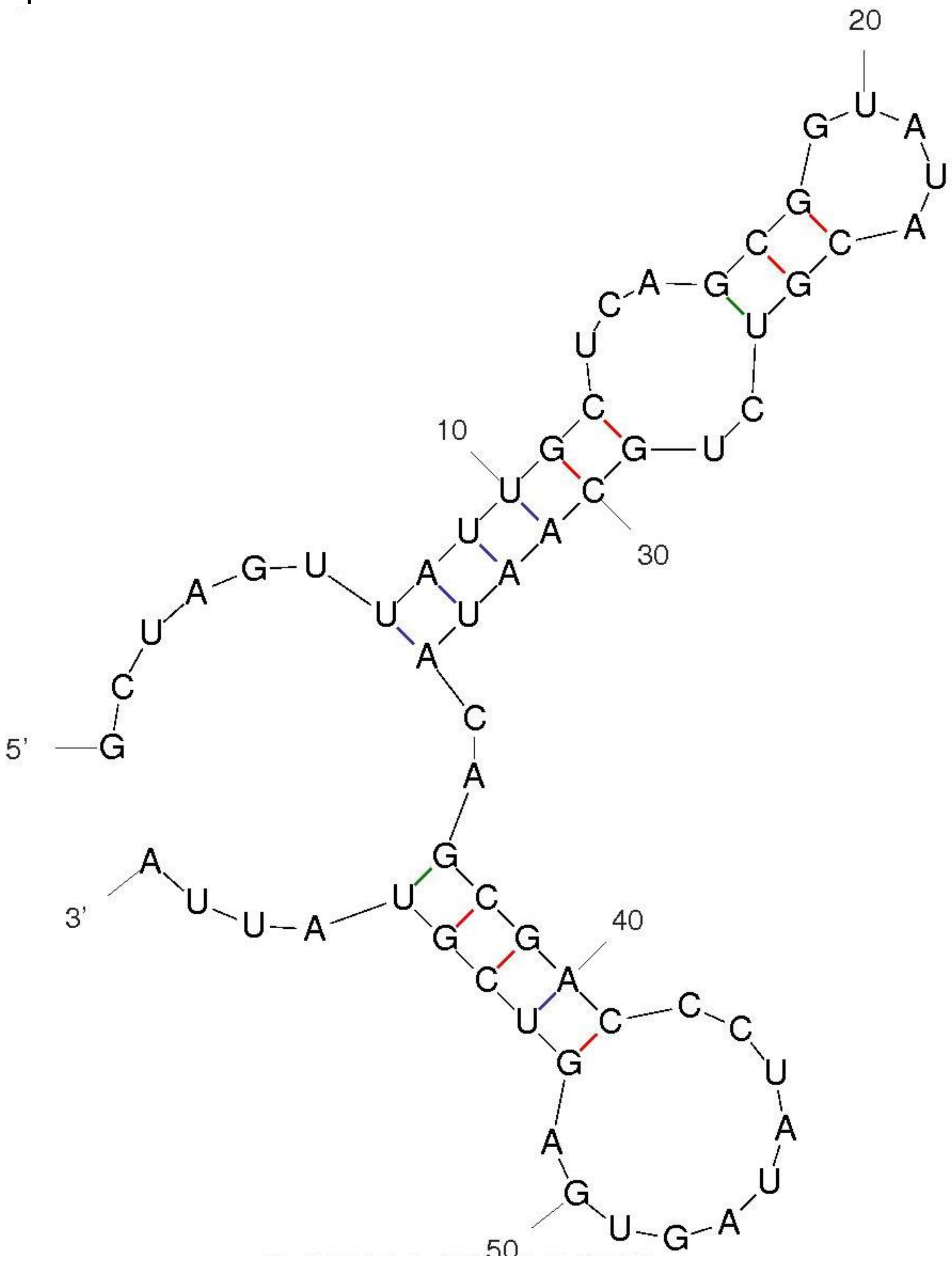

Temp4

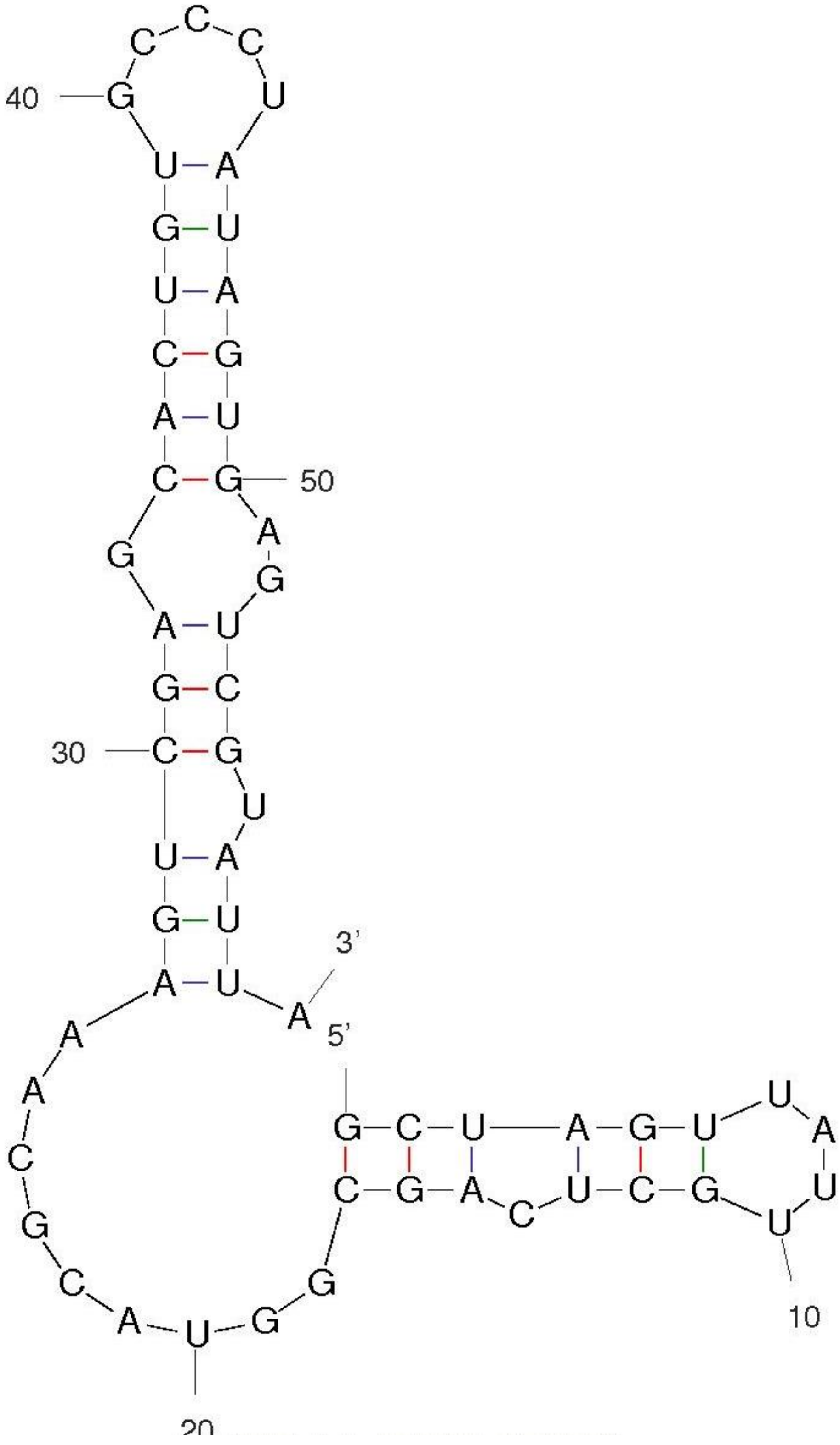

References

[1] Bolger, A. M.; Lohse, M.; Usadel, B. *Bioinformatics* **2014**, *30*, 2114–2120.
[2] Langmead, B.; Trapnell, C.; Pop, M.; Salzberg, S. L. *Genome Biol.* **2009**, *10*, R25.
[3] Zuker., M. *Nucleic Acids Res.* **2003**, *31*, 3406–3415.
